# Potential use of Indonesian basil (*Ocimum basilicum*) maceration to increase estradiol and progesterone synthesis and secretion to improve prenatal growth of offspring using female albino rats as an animal model

**DOI:** 10.14202/vetworld.2022.1197-1207

**Published:** 2022-05-18

**Authors:** Andriyanto Andriyanto, Leliana Nugrahaning Widi, Mawar Subangkit, Elpita Tarigan, Yusa Irarang, Rindy Fazni Nengsih, Wasmen Manalu

**Affiliations:** 1 Department of Anatomy, Physiology, and Pharmacology, Faculty of Veterinary Medicine, IPB University, Bogor, Indonesia; 2 Department of Clinic, Reproduction, and Pathology, Faculty of Veterinary Medicine, IPB University, Bogor, Indonesia; 3 Graduate School of Veterinary Biomedical Science, Faculty of Veterinary Medicine, IPB University, Bogor, Indonesia

**Keywords:** Indonesian basil, rat, reproductive system

## Abstract

**Background and Aim:**

Basil is well known as a medicinal plant that contains high essential oils and antioxidant compounds that have the potential to improve ovarian development. Thus, basil may have the potential to improve the growth and development of the uterus and placenta for optimal prenatal growth of offspring. This study aimed to evaluate the effect of Indonesian basil maceration on gonad development of mature female albino rats.

**Materials and Methods:**

Fifteen 8-week-old female Sprague-Dawley rats, at the diestrus stage of the estrus cycle, were divided into three different treatment groups: Control group (mineral water), bas-low group (1% of basil maceration), and bas-high group (5% of basil maceration). Basil maceration was dissolved and administered in mineral drinking water, and the treatments were given for 20 days (4 estrus cycles). At the end of the treatment period, serum follicle-stimulating hormone (FSH), estradiol, and progesterone (Pg) were measured using enzyme-linked immunosorbent assay. The relative weight of the ovary and uterus; diameter and length of uterine cornual; vascularization of uterus; the diameter of uterine glands; the number of primary, secondary, and tertiary de Graaf follicles; the number of corpora luteum; as well as the expression of vascular endothelial growth factor (VEGF) in the ovary were determined.

**Results:**

There was no significant difference (p>0.05) in the serum FSH level of rats treated with basil maceration drinking water doses of 1% and 5% compared to the control group. However, serum estradiol and Pg concentrations in the 1% and 5% basil maceration groups were significantly higher (p<0.05) than those of the control group. Furthermore, 1% and 5% basil maceration significantly increased the uterus’s relative weight, diameter, and vascularization. Serum estradiol concentrations contributed to the elevated expression of VEGF compared to Pg.

**Conclusion:**

Administration of basil maceration for 20 days before mating could improve follicle growth and development, eventually increasing estradiol synthesis and secretion, thus improving the uterus’s preparation for implantation. This makes basil maceration an attractive candidate in clinical research to enhance the growth and development of the uterus and placenta, which will better support the optimum prenatal growth and development of embryos and fetuses, resulting in superior offspring.

## Introduction

Plants have received significant attention for their medicinal properties, contributing to their usage as alternative and complementary drug therapies for various diseases, including reproductive health disorders [1]. Ettawa Grade flaxseed oil supplementation significantly stimulates the development of large preovulatory follicles, producing a higher number of twin births with a larger weight and higher birth weight, and eventually producing offspring with a larger weaning weight [2].

Basil (*Ocimum bacilicum*), an annual herb of the Lamiaceae family, is widely cultivated in Asia as a nutrient-rich food and herbal medicine [3]. Indonesian people consume the fresh and raw leaves of the basil plant, and the roots of basil are used in India as a medicine [4,5]. At present, basil is grown and used worldwide in the medical, food, pharmaceutical, and cosmetics industries [6]. The medicinal properties that basil contains can be leveraged as a therapeutic for various diseases, which will be beneficial for human health, especially reproductive health [6].

Biologically active compounds found in basil have been shown to have pharmacological activities that can be used to treat numerous conditions [7]. Basil leaf extract has several beneficial properties, such as being an estrogenic [8], antihyperlipidemic [9,10], antioxidant [11,12], and anti-hypoglycemic [13]. In addition, basil extracts can potentially be used to eliminate pathogenic bacteria [14]. The antimicrobial effects of basil can also be used to treat and prevent reproductive tract infections, which are generally caused by bacterial infections [15]. Furthermore, basil leaf ethanol extract is helpful in treating the clinical and pathological abnormalities of polycystic ovarian syndrome, preventing ovarian cell dysfunction, increasing fertility, and significantly increasing uterine weight [8]. Furthermore, β-sitosterol and stigmasterol, found in plants, significantly increased implantation, and uterine weight [16,17]. In addition, basil is a rich store of chemicals, such as eugenol, which can be used to treat cancer in reproductive organs [18,19]. Moreover, Gautam *et al*. [1] reported that basil had no toxic effect on the reproductive system. Consuming lemon basil can also enhance the excretion of toxic substances safely from the body [20], which is especially important for pregnant women.

Based on the positive effects of basil, the plant has a great potential to be used to improve body conditions prior to pregnancy, which will improve health conditions during pregnancy. A healthy body condition before mating will provide better support for the prenatal and postnatal growth of the offspring. Therefore, the present study aims to evaluate the effect of Indonesian basil maceration on female albino rat gonad development as an experimental model.

## Materials and Methods

### Ethical approval

The use of experimental animals in this study has been approved by the Animal Ethics Committee Faculty of Veterinary Medicine, Bogor Agricultural University with registration number: 007/KEH/SKE/I/2020.

### Study period and location

The study was conducted in August and September 2021. The study was conducted in IPB University and iRATco laboratory services, Bogor, Indonesia. The animals were acclimatized for seven days and followed by experimental treatment for 20 days. The animals were kept in individual ventilated cages unit (Slimeline Plus, Tecniplast, Italy) with a temperature range between 22-25°C and relative humidity range between 45-65% during the experiment.

### Experimental design

A total of 15 female, 8-week-old Sprague-Dawley rats (iRATco Veterinary Laboratory Services, Indonesia) weighing around 200-230 g in the diestrus phase of the estrus cycle were divided equally into three different groups. The first group was the control group consisting of rats given mineral drinking water. The second group was the bas-low group, consisting of rats given 1% basil maceration in drinking water, and the third group was the bas-high group, consisting of rats given 5% basil maceration in drinking water. The treatments were given for 20 days (four consecutive estrus cycles). The experimental rats were administered with basil maceration for 20 days. Each experimental group was given 200 mL of drinking water containing basil maceration during the treatment (0, 1, and 5%) twice daily, every 12 h. After 20 days of treatment, all experimental rats were euthanized using ketamine (100 mg/kg of body weight [BW]) (Ket-A-100^®^, Agrovet Market, Canada) and xylazine (3 mg/kg of BW) (Xyla, Interchemie, Netherlands), followed by perfusion as described previously by Ko *et al*. [21]. The experimental rats were maintained by following the standard guideline for animal care and use [22] with *ad libitum* standard rodent diet for feeding and drinking water access.

### Preparation of Indonesian basil maceration

The basil plant *Ocimum basilicum* was collected from Bogor, West Java, Indonesia, from the same source described previously by Hikmawanti *et al*. [23]. We used the leaves part in this study. The maceration was prepared by adding 1000 mL water to 500 g (50% w/v) of fresh Indonesian basil and boiled at 100°C for 30 min as a maceration stock. A drinking solution of basil maceration at a dose of 1% was prepared by adding 2 mL of maceration stock to 98 mL of drinking water to prepare 100 mL as final volume. The 5% basil maceration drinking water was prepared by adding 10 mL of maceration stock to 90 mL of drinking water to prepare 100 mL final volume. Basil maceration drinking water was freshly prepared by diluting the stock solution with mineral water.

### Follicle-stimulating hormone (FSH), estradiol, and progesterone (Pg) assays

Three milliliters of intracardial blood were collected into a plain vacutainer and allowed to clot without anticoagulant. The serum was separated by centrifugation (1500× *g*, 20 min, 4°C) and stored at −20°C until analyzed. The concentrations of FSH, estradiol, and Pg were determined using Rat FSH enzyme-linked immunosorbent assay (ELISA) Kit (Elabscience, Cat no. E-EL-R0391, US), Rat/Porcine E2 (Estradiol) ELISA Kit (Elabscience, Cat no. E-EL-0152, US), and Pg ELISA Kit (Elabscience, Cat no. E-EL-0154, US) according to the manufacturer’s instructions and read the optical density by ELISA reader (TC 96 microplate reader, Teco Company, Canada).

### Uterine analysis

After 20 days of treatment, before euthanasia, a picture of the uterus was taken by Olympus stereo microscope SD30 (Olympus, Japan) and Canon EOS D1100 SLR camera (Canon, Taiwan) for vascular analysis for ideal and live vascular conditions. The length of the left and right uterine cornua, the diameter of the uterine cornua, and the proportion of the uterine blood vessel area with total uterine area were analyzed using ImageJ software (National Institutes of Health, US) and the relative weight of the uterus was measured by weighing the uterine and BW. The proportion of uterine blood vessels was calculated using the following formula.


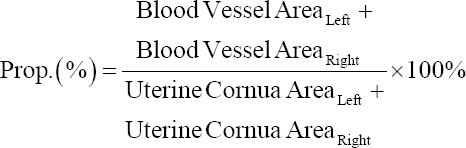


The relative weight of the uterus was calculated using the following formula.


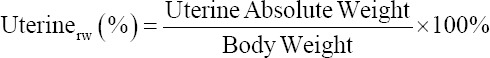


### Ovarian analysis

The total number of superficial follicles was calculated manually under the light stereomicroscope (Olympus, Japan) in the right and left ovaries. The sizes of follicles were measured using image analysis methods in ImageJ (NIH, US). The relative weight of the ovary was measured, similar to the method used to measure the relative weight of the uterus.


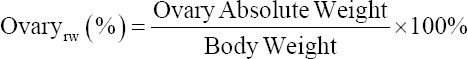


### Histology

The ovary and uterine tissues were fixed in 10% buffered formalin and then dehydrated and embedded in paraffin. The paraffinized tissue blocks were cut into 4 μm thick sections using a Leica RM2135 Microtome (Leica, Germany). After assembly into a glass slide, the tissue was deparaffinized in xylene, rehydrated in graded ethanol, and submitted to hematoxylin and eosin (H&E) staining and sealing with Entellan® (Merck Millipore, Germany). The H&E slides were then reviewed and examined using a light microscope (Primostar, Zeiss, US).

### Immunohistochemistry

Immunohistochemical staining for vascular endothelial growth factor (VEGF) in the ovary was conducted using the immunoperoxidase activity technique. Poly-l-lysine coated glass slides (Biogear, Indonesia) were used. Parrafin embedded sections were dewaxed, rehydrated, and subjected to quenching of endogenous peroxidase activity. Antigen retrieval was enhanced using HIER solution epitope retrieval (Cat. no. CPL500, ScyTek, US) for 5 min at 121°C. Sections were washed with phosphate buffer saline 10 mM for 5 min with pH 7.4, followed by incubation with a specific antibody to VEGF. Sections were incubated with a mouse monoclonal antibody to VEGF at 4 μg/mL concentration (sc-7269; Santa Cruz Biotechnology, US), then followed by immersion in wash buffer for 5 min and visualization with polymer EnVision Flex™ (Dako Omnis, Agilent, US).

### Data visualization and statistical analysis

Data visualization in the box plot, bar plot, and statistical analysis were performed in R software ver. 3.5.1 (www.r-project.org). Normal distribution data analysis was calculated using the Shapiro-Wilk test and equality of variance was calculated using the Bartlette test. The data expressed as mean±standard deviation were analyzed by one-way analysis of variance followed by Tukey *post hoc* test. p<0.05 was considered statistically significant.

## Results

The rats were in good physiological condition and showed no toxicity after the 20-day treatment with basil maceration drinking water at all doses. All rats developed well with high appetites, active locomotion, and no abnormal appearance. There was no damage to the liver and kidney after the treatment by histology. Hematology as well as liver and kidney functions showed that the experimental rats were in the normal range, and there was no significant difference in parameters between the groups (data not shown).

### Relative weights of the uterus and ovary

**Figure-1:**
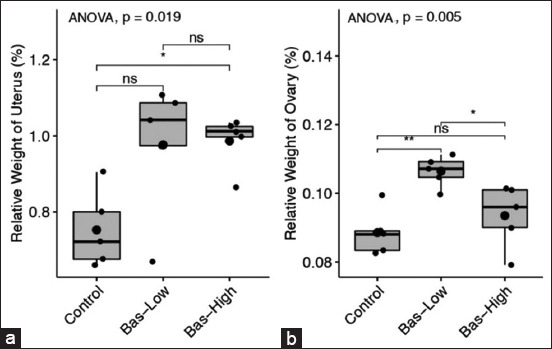
(a and b) Relative weight of uterine (left) and ovary (right) comparison after 20 days application of 1% Basil (bas-low) and 5% Basil (bas-high) in albino rat. Statistical analysis using analysis of variance and *post hoc* Tukey-honestly significant difference with NS=p>0.1; ns=p>0.05; *=p<0.05; **=p<0.001; and ***=p<0.0001.

The relative weights of the uterus and ovary are presented in Figure-1. The relative weights of the uteri in the 1% basil maceration (bas-low) experimental group were statistically similar to those in the control group (p>0.05). However, the 5% basil maceration (bas-high) experimental group had significantly higher relative weights of the uterus compared to those of the control group (p<0.05). There was no difference in the relative weight of the uterus between the bas-low and bas-high groups (p>0.05). In the bas-low group, one out of the five rats showed a low relative uterine weight, which was similar to the control rat uterine weights. However, the mean relative weights of the uteri from the bas-low group were similarly high compared to those from the bas-high group. In contrast, the relative weight of the ovaries of bas-low group showed significantly higher (p<0.05) than those of control and bas-high groups. The range of relative weights of the ovaries was extensive (Figure-1b).

### Uterine analysis

**Figure-2:**
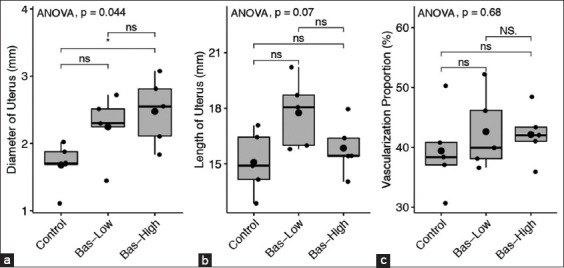
(a-c) Macroscopy analysis in uterine comparison between control with 1% (bas-low) and 5% (bas-high) of Basil maceration in albino rat. Statistical analysis using Analysis of variance and *post hoc* Tukey-honestly significant difference with NS=p>0.1; ns=p>0.05; *=p<0.05; **=p<0.001; and ***=p<0.0001.

**Figure-3:**
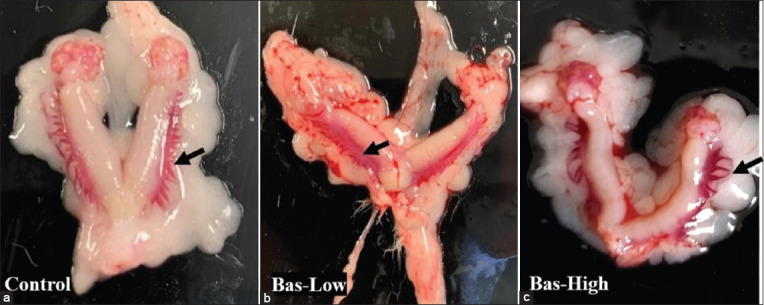
(a-c) Gross appearances and vascularization of the uteruses of albino rats treated with basil maceration at doses of 0% (control), 1% (bas-low), and 5% (bas-high). Bar: 10 μm.

The diameters of the uterine cornua from the bas-high group were significantly higher (p<0.05) compared to the diameters of the control rats. However, the diameters of uterine cornua of the rats from the bas-low and bas-high were statistically similar (p>0.05). Moreover, the diameters of uterine cornua of rats from the bas-low groups were statistically similar (p>0.05) to the diameters from the control rats even though the mean diameters in the low-bas group were numerically higher (Figure-2a). This non-significant difference is due to the high variations of the diameters of the uteri. One uterus from the control group and one from the bas-low group had a similarly low diameter of uterine cornua (Figure-2a). In contrast with the diameter of uterine cornua, the length of uterine cornua and the proportion of vascularization in the uterine cornua from rats in the bas-low and bas-high were similar to those in the control experimental rats without basil maceration (Figure-2b and c). This is due to the high variations of the values in the expressed parameters. Two out of five rats in the bas-low group and four out of five rats in the bas-high group had uterine cornua lengths similar to the lengths of the control groups. In proportion of uterine vascularization data, 4/5 bas-low rats and 5/5 bas-high rats were in similar ranges compared to control values (Figure-2), as shown in macroscopy data in Figure-3.

**Figure-4:**
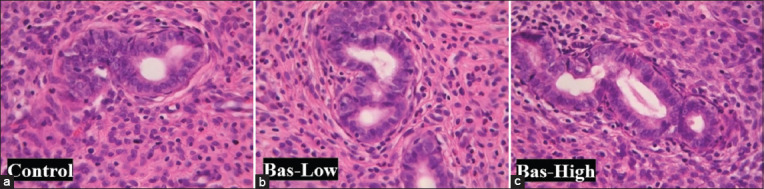
(a-c) Histological appearances of the uteruses of albino rats treated with basil maceration at doses of 0% (control), 1% (bas-low), and 5% (bas-high). Hematoxyline and Eosin. Magnification 400×, Bar: 200 μm.

**Figure-5:**
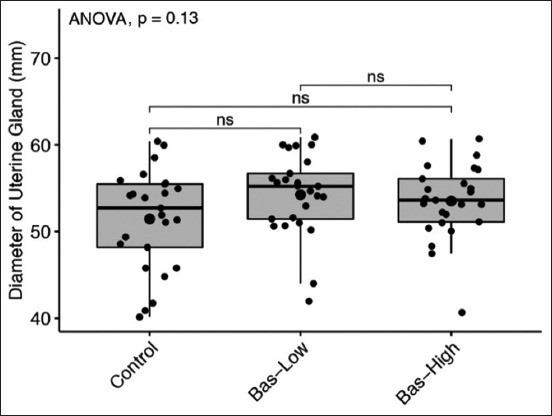
Diameter of uterine gland comparison between control with 1% (bas-low) and 5% (bas-high) of Basil maceration in albino rat. Statistical analysis using analysis of variance and *post hoc* Tukey-honestly significant difference with NS=p>0.1; ns=p>0.05; *=p<0.05; **=p<0.001; and ***=p<0.0001.

In line with the macroscopy data in Figure-3, histopathology data of uterine glands showed that bas-low and bas-high rats had higher gland activities, as indicated in the size of the uterine gland (Figure-4). However, the uterine gland diameters between the experimental groups were not statistically different. All bas-low and bas-high rats had uterine gland diameters that were not significantly different from the control group (Figure-5).

### Ovarian follicle number

**Figure-6:**
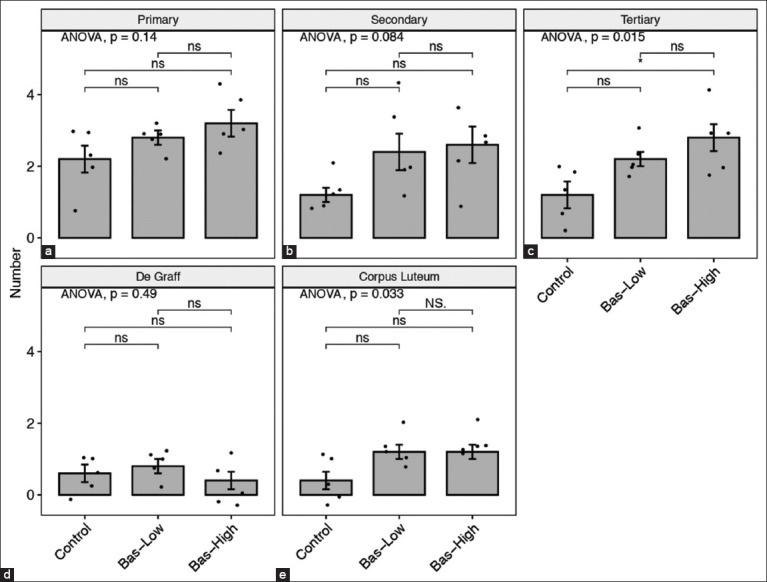
(a-e) The number of presence ovarian follicle comparison between control with 1% (bas-low) and 5% (bas-high) of Basil maceration in albino rat. Statistical analysis using analysis of variance and *post hoc* Tukey-honestly significant difference with NS=p>0.1; ns=p>0.05; *=p<0.05; **=p<0.001; and ***=p<0.0001.

**Figure-7:**
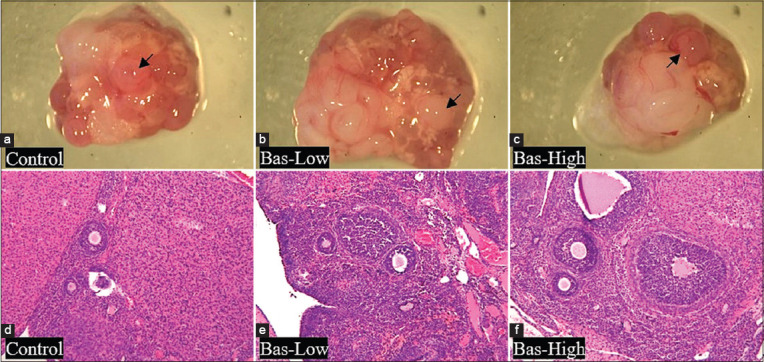
Macroscopy (a-c) and microscopy (d-f) of the ovaries of albino rats treated with basil maceration at doses of 0% (control), 1% (bas-low), and 5% (bas-high). Macroscopy (a-c) Bar: 5 mm and Microscopy (d-f). Microscopy staining: Hematoxylin and Eosin, Magnification 100×, Bar: 400 μm.

The number of tertiary ovarian follicles in the bas-high rats was significantly higher (p<0.05) than in the control rats. However, there was no significant difference (p>0.05) in the number of tertiary ovarian follicles between the bas-low and bas-high rats and between the bas-low and control rats. In contrast, there was no significant difference observed in the numbers of primary follicles, secondary follicles, follicles de Graaf, and corpora lutea among all groups. A total of 3/5 rats in the bas-low group and 4/5 samples in the bas-high group showed higher numbers of corpora lutea than the control group, which is in line with the primary and secondary follicle data (Figure-6). Macroscopy analysis showed that bas-low and bas-high had more mature follicles than the control rats (Figure-7a-c). Rats treated with basil maceration had more active mature follicles (arrow) than control rats. In the histopathology analysis, there was no significant difference in the quality of follicles in the ovaries of all the groups (Figures-7d-f). Microscopic evaluation of the ovaries (Figures-7d-f) showed that there were no significant microscopic changes in the ovaries between the groups.

### The expression of VEGF protein in the uterus

**Figure-8:**
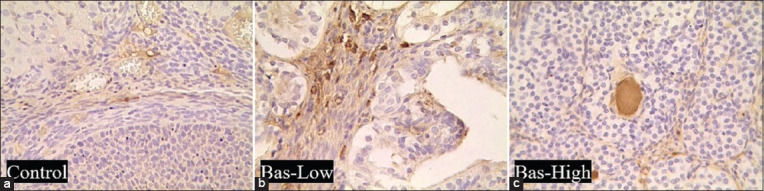
(a-c) Vascular endothelial growth factor expression with immunohistochemistry staining in the ovaries of albino rats treated with basil maceration at doses of 0% (control), 1% (bas-low), and 5% (bas-high). Immunohistochemistry, DAB and Hematoxylin counterstain. Magnification 400×, Bar: 200 μm.

**Figure-9:**
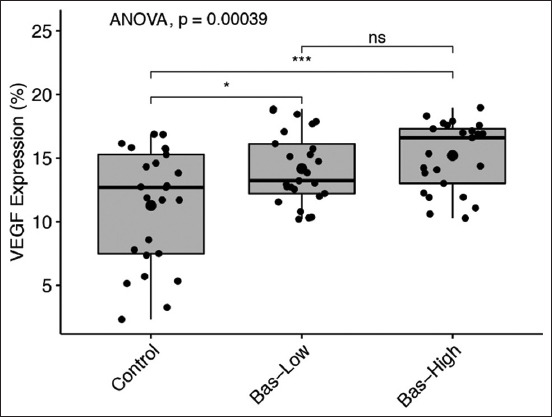
Vascular endothelial growth factor (VEGF) expression quantification data by Image analysis. Bas-low and bas-high showed higher significance (p<0.05) in VEGF expression than Control group. Statistical analysis using analysis of variance and *post hoc* Tukey-honestly significant difference with NS=p>0.1; ns=p>0.05; *=p<0.05; **=p<0.001; and ***=p<0.0001.

The expression of VEGF located in the interstitial tissue of the ovaries indicated that neovascularization is in progress (Figure-8). Bas-low and bas-high rats showed significantly higher (p<0.05) VEGF expression compared to the control. However, no significant difference was observed in VEGF expression between basil maceration experimental groups (Figure-9).

### Serum concentrations of FSH, estradiol, and Pg

**Figure-10:**
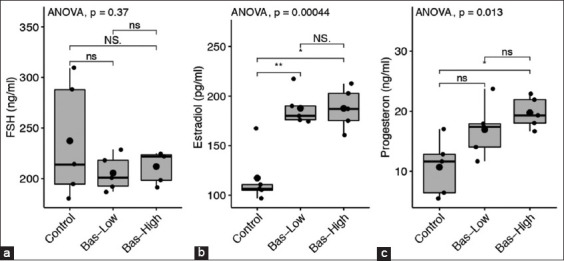
Follicle-stimulating hormone (a), Estradiol (b) and Progesterone (c) serum level concentration comparison between control with 1% (bas-low) and 5% (bas-high) of Basil maceration in albino rat. Statistical analysis using analysis of variance and *post hoc* Tukey-honestly significant difference with NS=p>0.1; ns=p>0.05; *=p<0.05; **=p<0.001; and ***=p<0.0001.

There was no significant difference observed in the concentrations of FSH in bas-low and bas-high rats compared to the control group (Figure-10a). However, all rats (5/5) in both the bas-low and bas-high groups were lower than the mean of the FSH concentration in the control group. In contrast, the concentration of estradiol in the bas-low and bas-high rats was significantly higher (p<0.05) than the mean concentration in the control rats without basil maceration treatment (Figure-10b). In addition, the concentration of Pg was significantly higher in bas-high rats than that in the control rats. Three rats in the bas-low group had a higher Pg mean concentration compared to the highest value in the control group (Figure-10c).

**Figure-11:**
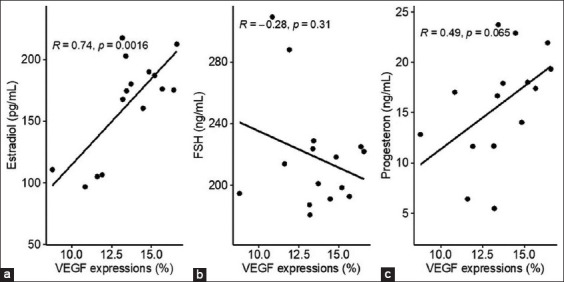
(a-c) Correlation between vascular endothelial growth factor expressions with serum estradiol, follicle-stimulating hormone, and progesterone concentrations in the experimental albino rats.

There was a negative correlation between FSH and VEGF expression (R=−0.28) (Figure-11). High expression of VEGF suggested that more FSH was metabolized; thus, this reduced FSH circulation in the periphery. A moderate positive correlation value (R=0.74) was observed between estradiol and VEGF expression to form vascularization, and Pg had a low positive correlation with VEGF expression to form vascularization (R=0.49).

## Discussion

In this study, the administration of basil maceration in mature female Sprague-Dawley rats, at the diestrus phase of the estrus cycle, for 20 days showed the potential for basil maceration to improve the health, gonadal growth, and development of female animals. Optimal growth and development of ovaries will result in high-quality follicles that will produce high concentrations of estradiol and Pg. Estradiol and Pg are reproductive hormones that will stimulate the growth and development of the uterus and placenta to prepare and provide the ideal environment to support the embryos and fetus during pregnancy [24]. The optimal environment during prenatal development will induce a more favorable gene set expression and improve the postnatal life of the offspring. Therefore, basil has the potential to be used to improve reproductive performance, resulting in better outcomes for offspring.

In this study, the stages of gonad development and estrus cycles of the experimental rats were different because the estrus cycles were not synchronized. We chose the stage of diestrus during the selection of the experimental rats before the treatment. The treatments were given for 20 days, or around four consecutive estrus cycles, and the experimental parameters were evaluated at the end of the treatment period. Although the treatment was started at the diestrus phase, most of the duration of the treatment was in the estrus cycle, which can vary between each rat. Thus, at the end of the 20-day treatment, the phases of estrus cycles of the experimental rats were not in the same stage, which led to a high variation in the gonad developments of experimental rats. Nevertheless, the significant effects of basil maceration supplementation on gonad development could still be observed.

There have been limited studies on the use of basil to improve reproductive performances in males [3,25] and female animal reproduction [4,8]. In this study, we show that experimental rats treated with 1% basil maceration (bas-low) experience an increase in the growth and development of gonads, reflected in the non-significant increase in the relative weight of the uterus. However, the experimental rats treated with 5% basil maceration (bas-high) showed a significant increase in uterine relative weight. The non-significant increase in the relative weight of the uterus in the experimental rats treated with 1% basil maceration is due to the high variation in the relative weights of the uteri in the experimental rats. In addition, the non-significant effects of treatment of basil maceration on the relative weight of the ovaries were attributed to the similarly high variation in all treatment groups. Although non-significant, the increasing weights of the ovaries in the bas-low rats are related to the activities of follicles growth and developments in the ovaries to produce ovum. In contrast, the increasing weight of the uterus is related to improved hormonal stimulation by ameliorating estradiol and Pg secretion.

The physiological processes caused the increases in uterine and ovary weights in those organs. The present study shows that experimental rats treated with basil maceration have higher estradiol and Pg, stimulating uterine tissue growth. The ovaries produce essential hormones in female reproductive organs, such as estradiol and Pg, which can boost the proliferation of the uterus and increase uterine weight. In the previous studies, the administration of estradiol in rodents induced uterine growth, particularly in the endometrium, and the growth affected the bioavailability of estradiol in serum [26,27]. These studies demonstrated that sufficient amounts of exogenous estradiol induce endometrial growth and maturation in rats, as long as estradiol levels remain high in the circulation for a long duration. In our study, serum estradiol levels produced by endogenous secretion from the ovarian follicle were remarkable, resulting in higher uterine weight.

In line with the results, the leaf extract of basil showed activity, such as estrogenic [8], which can stimulate uterine proliferation. The uterus also produces prostaglandin (PGE2). Prostaglandin (PGE2) is a potent vasodilator [28] that can promote active vascularization, experience local edema, and increase uterine weight [29]. The ovaries of experimental rats treated with basil maceration showed many mature follicles. The mature follicles are caused by the presence of FSH secreted by the pituitary that stimulates the growth and maturation of follicles. Treatment of mice with lemon basil leaves was reported to increase the synthesis and secretion of FSH and estradiol [4]. Basil treatment showed the effects of elevating the metabolism of circulatory FSH by increasing the activity of follicles growth and development in the ovary. In this study, the FSH concentration of rats treated with basil maceration is lower than that of control rats without basil maceration treatment. The data indicated that FSH was used to stimulate the growth of follicles in the ovary. Thus, serum FSH concentration became low. FSH interacted with ovarian FSH receptors in the ovary to mediate follicular development and estradiol synthesis [30].

Growing follicles produce estradiol, and Pg is a hormone produced by the corpus luteum in the ovaries after ovulation of the follicles and during early pregnancy [31]. The concentrations of estradiol and Pg in the experimental rats treated with basil maceration were significantly higher compared to the control rats. The increased estradiol concentrations will stimulate the growth and development of uterine glands. Pg is produced by the corpus luteum and is responsible for preparing the uterus for implantation and maintaining pregnancy in pregnant rats [32].

The increase in estradiol concentration following basil maceration is correlated with improved growth and development of follicles. High-quality follicles transform into a high-quality corpus luteum. Therefore, this improves the Pg synthesis and secretion that will stimulate the growth and development of the uterine glands in preparation for pregnancy, even though the experimental animals were not mated. The basil treatment-induced increase in estradiol secretion by the growing follicles and the increased Pg synthesis and secretion by the corpus luteum will result in increased Pg concentration in the plasma, stimulating the expression of VEGF to encourage uterine vascularization [33]. This experiment showed that higher estradiol and Pg concentrations in the experimental rats treated with basil maceration experienced greater vascularization of the uterus by increasing the expression of VEGF, which was confirmed by the strong positive correlation between estradiol and Pg concentrations with ovarian VEGF expression. High expression of VEGF in organs indicates high circulating VEGF [34].

These hormones will also stimulate the growth and development of uterine glands to produce nutrients, materials, and growth factors to provide the optimal environment to support the development of embryos. Our study showed that rats subjected to basil maceration treatment underwent better gland development. The previous study mentioned that uterine function is primarily regulated by the ovarian steroid hormones estrogen and Pg [35]. Elevated estradiol levels have several important roles, such as the proliferation of endometrium and cervical mucus production, augmentation of gonadotropin action, and the acceleration of steroidogenesis [36]. The functions of estrogen extend to maintaining and improving the luminal epithelium, glandular epithelia, and stroma by expressing genes important for uterine receptivity and blastocyst implantation [37]. The improved development of the uterine gland results in higher production of essential hormones, which ultimately affects fetal survival and growth, uterine receptivity, embryo implantation, stromal cell decidualization, and placental development [38,39].

Intensive and active vascularization in the uterus improves the transport of nutrients, oxygen, and growth factors to support the future growth and development of embryos and fetuses. However, in this study, the experimental rats were not mated, and the production of materials needed to support implantation still occurred. The intensity of uterine vascularization in the experimental rats treated with basil maceration was greater than in those without basil treatment. The increase in uterine vascularization indicates that the uterus is in the active phases. As observed in the correlation analysis, the increasing concentrations of estradiol are followed by the active vascularization, supported by the increased VEGF expression. Improvement of vascularization by estradiol and Pg in the uterus will result in the optimization of nutrients, oxygen, and other molecules required by the growing embryos, and thus will improve the genetic expression in the embryos.

Our previous work has shown that the increase in estrogen and Pg concentrations during pregnancy will improve prenatal growth, and eventually improved birth weight in lambs [40]. The increased maternal serum Pg concentrations by application of the superovulation technique improved the uterine and fetal weights at weeks 7 and 15 of pregnancy in Javanese thin-tail ewes [41]. In addition, serum Pg concentration had a strong correlation with uterine and fetal weights at weeks 7 and 15 of pregnancy in Javanese thin-tail ewes, confirming that the increased Pg synthesis and secretion during pregnancy will improve uterine growth and development to better support embryonic and fetal growth and development [42]. Therefore, lamb birth weight correlated with the concentrations of hormones and metabolites in the maternal serum during pregnancy [43].

Increasing estrogen and Pg concentration during pregnancy by super ovulating the ewes before mating significantly increased lambs’ birth weights and preweaning weights [44]. The increased lamb birth weights in the superovulated ewes before mating also improved preweaning weights of the birth lambs, because the increased estrogen and Pg during pregnancy stimulated the growth and development of mammary glands that dramatically increased milk production to support the growth and development of lambs during the preweaning period [45]. The improved uterine environment and the optimum condition during prenatal growth will optimize the genetic expressions of the birth lambs that will improve the growth and survival of the offspring during postnatal life. Since the superovulation by using commercial gonadotropin hormone is expensive and has a side effect of increased litter size, our team also designed the optimum dose for stimulating the endogenous secretion of Pg and estradiol without increasing litter size in Kacang goats [46].

The improved uterine environment by increasing estradiol and Pg hormones during pregnancy by superovulation significantly improved the expression of growth hormone genes in the offspring that can improve the growth performance of the offspring during postnatal life [47]. The application of this technology to increase the uterine environment during prenatal life significantly enhances the immunity and health condition of the offspring, as indicated by the improved lambs’ resilience to *Haemonchus contortus* [48]. Therefore, the improved prenatal environment by elevated synthesis and secretions of reproductive hormones using basil maceration confirms that the basil herb is very prospective to be used and developed to improve the reproductive performances and the quality of offspring produced.

Basil maceration can increase estradiol and Pg synthesis and secretions. This material is prospective to be developed to improve endogenous secretions of pregnancy hormones (estradiol and Pg) in mammalian animals to improve reproductive performances and the quality of offspring produced so that they have good growth performances with a better capacity to survive by enhancing the growth and health conditions. In addition, it is prospective to combine several herbal materials in combination with gonadotropin stimulation of follicles growth and development to optimize the production of hormones, growth factors controlling and regulating the uterus and placental growth, development to support the optimum growth, and development of embryos and fetuses during prenatal growth during pregnancy.

## Conclusion

Basil maceration application for 20 days (four estrus cycles) in albino rats before mating improves the ovary’s growth and development, eventually increasing estradiol and Pg synthesis and secretions that ultimately improve the growth and development of the uterine tissues during the premating period. The optimum growth and development of the uterine environment during pregnancy will optimize the genetic expression of the embryos and fetuses that eventually produce offspring with better growth performances and health conditions during preweaning and adult life.

## Authors’ Contributions

AA and WM: Designed the study. LNW, MS, ET, YI, and WM: Conducted literature search. MS, ET, and YI: Conducted the experiment, data collection, and analysis. AA, MS, and WM: Conducted data acquisition. MS, ET, and YI: Conducted data analysis. LNW, MS, ET, YI, and RFN: Drafted and revised the manuscript. All authors have read and approved the final manuscript.
